# Supplementary material for: NOX2-Induced High Glycolytic Activity Contributes to the Gain of COL5A1-Mediated Mesenchymal Phenotype in GBM
Source: Cancers (Basel). 2022 Jan 20;14(3):516. doi: 10.3390/cancers14030516 (PMC8833670; doi:10.3390/cancers14030516)
Supplement: Supplementary file 1 [file cancers-14-00516-s001.zip › cancers-1540096-supplementary.pdf]

# Supplementary information

# NOX2-induced high glycolytic activity contributes to the gain of COL5A1-mediated mesenchymal phenotype in GBM.

YoungJoon Park [1,†], Min Woo Park [2,†], Junhyung Kim [2], Ju Won Ahn [1], JeongMin Sim [1], Ji-In Bang [3], Jinhyung Heo [4], Hyejeong Choi [5], Kyunggi Cho [1], Mihye Lee [2,*], Jong-Seok Moon [2,*] and Jaejoon Lim [1,*]

**Supplementary Table S1 - S2 and Supplementary Figure S1 – S9**

**Supplementary Table S1** Cohort I patients information

| Patient | Diagnosis | Grade | Age | Sex | Ki-67 | IDH mutation | 1p19q co-deletion | MGMT methylation | Overall survival (Month) |
|---|---|---|---|---|---|---|---|---|---|
| P1 | ganglioglioma | 1 | 28 | M | 1% | Wild-type | negative | negative | 56+ |
| P2 | ganglioglioma | 1 | 45 | M | 5% | Wild-type | negative | negative | 30+ |
| P3 | oligodendroglioma | 2 | 40 | M | 5% | Mutation | positive | negative | 78+ |
| P4 | diffuse astrocytoma | 2 | 33 | M | 5% | Wild-type | negative | negative | 69+ |
| P5 | diffuse astrocytoma | 2 | 75 | F | 7% | Wild-type | negative | negative | 18 |
| P6 | anaplastic astrocytoma | 3 | 34 | M | 5% | Wild-type | negative | positive | 5 |
| P7 | anaplastic astrocytoma | 3 | 56 | F | 20% | Wild-type | negative | negative | 19 |
| P8 | anaplastic oligodendroglioma | 3 | 46 | M | 10% | Mutation | positive | positive | 86+ |
| P9 | glioblastoma | 4 | 37 | F | 20% | Wild-type | negative | positive | 27+ |
| P10 | glioblastoma | 4 | 70 | F | 30% | Wild-type | negative | positive | 9 |
| P11 | glioblastoma | 4 | 59 | F | 10% | Wild-type | negative | positive | 26+ |

+; Alive, M; Male, F; Female, MGMT; O-6-methylguanine DNA methyltransferase

**Supplementary Table S2** Cohort II patients information

| Patient | Diagnosis | Grade | Age | Sex | Position |
|---|---|---|---|---|---|
| P1 | astrocytoma | 1 | 29 | F | Right frontal lobe |
| P2 | astrocytoma | 2 | | | Right frontal lobe |
| P3 | astrocytoma | 3 | 41 | M | Right frontal lobe |

M; Male, F; Female,

## Supplementary Figure S1

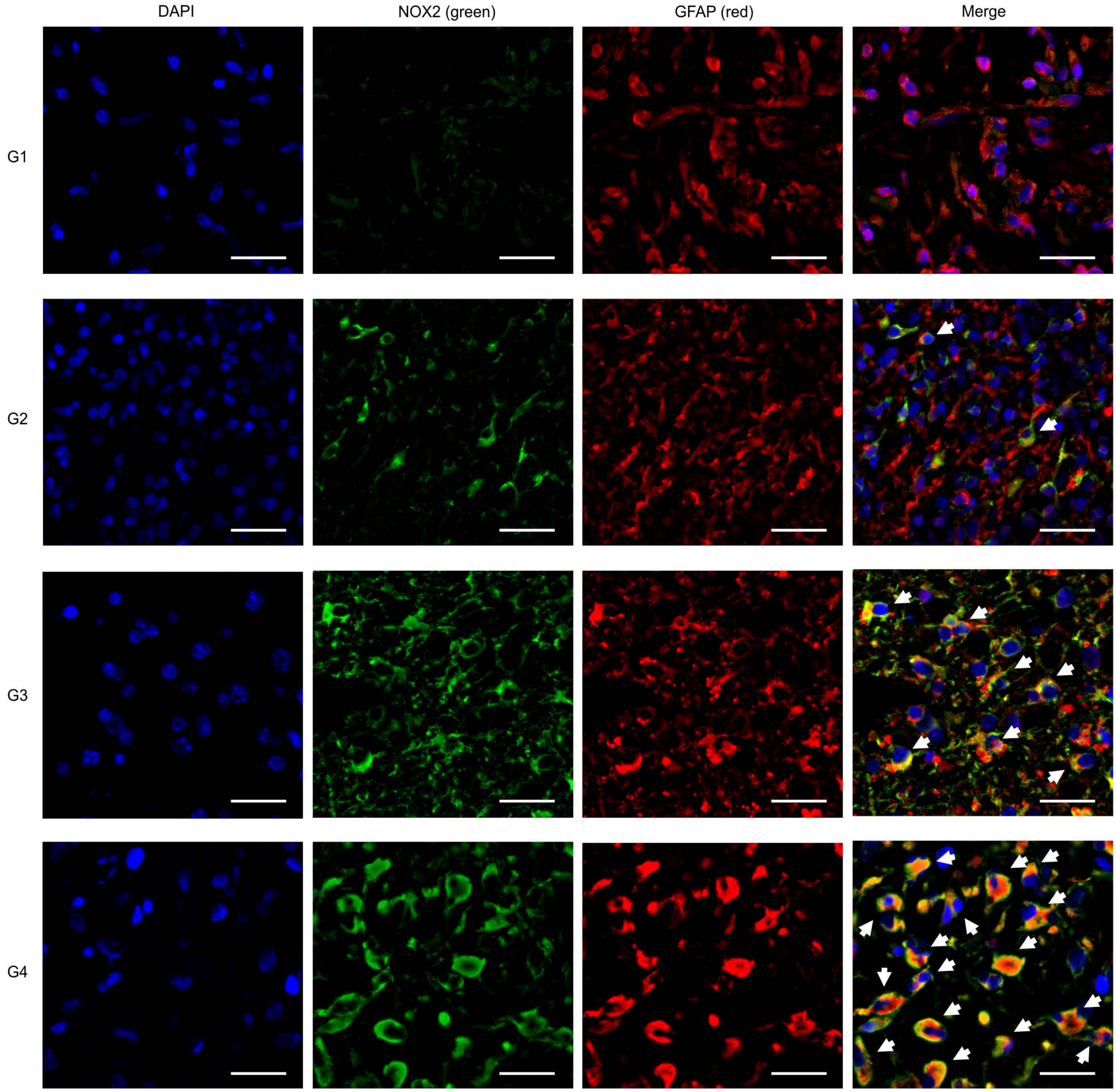


**Supplementary Figure S1.**

**The levels of NOX2 were elevated in patients with GBM.** Representative immunofluorescence images of NOX2 protein expression in tissues from patients with GBM (G4), G3 glioma (G3), G2 glioma (G2), and G1 glioma (G1) showing NOX2 (green) and astrocytes marker GFAP (red). DAPI-stained nuclei are shown in blue. Scale bars, 20 μM. White arrows indicate NOX2 and GFAP positive cells.

## Supplementary Figure S2

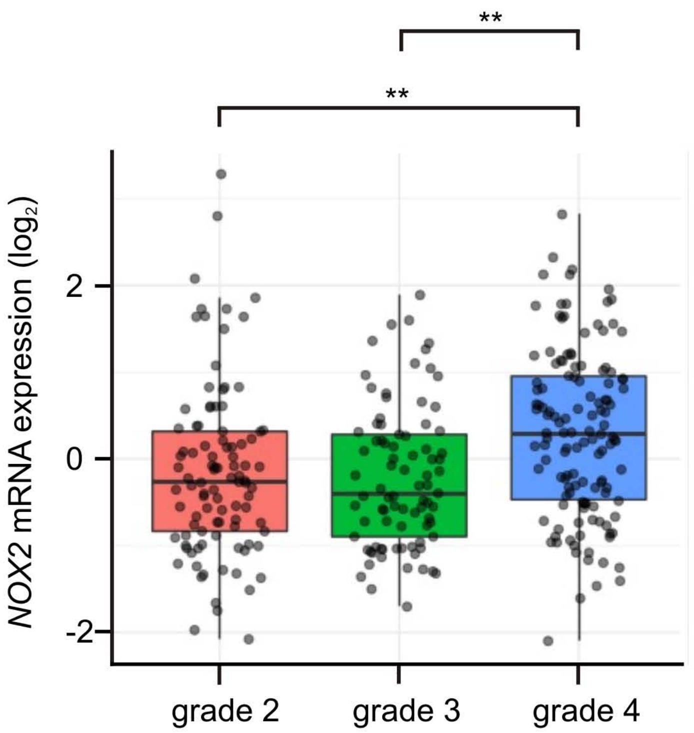


**Supplementary Figure S2.**
**Replication study for *NOX2* gene expression with REMBRANDT glioma dataset to evaluate results from TCGA.** The levels of NOX2 mRNA in patients with grade 2, grade 3 and grade 4 glioma. ** $p < 0.01$.

# Supplementary Figure S3

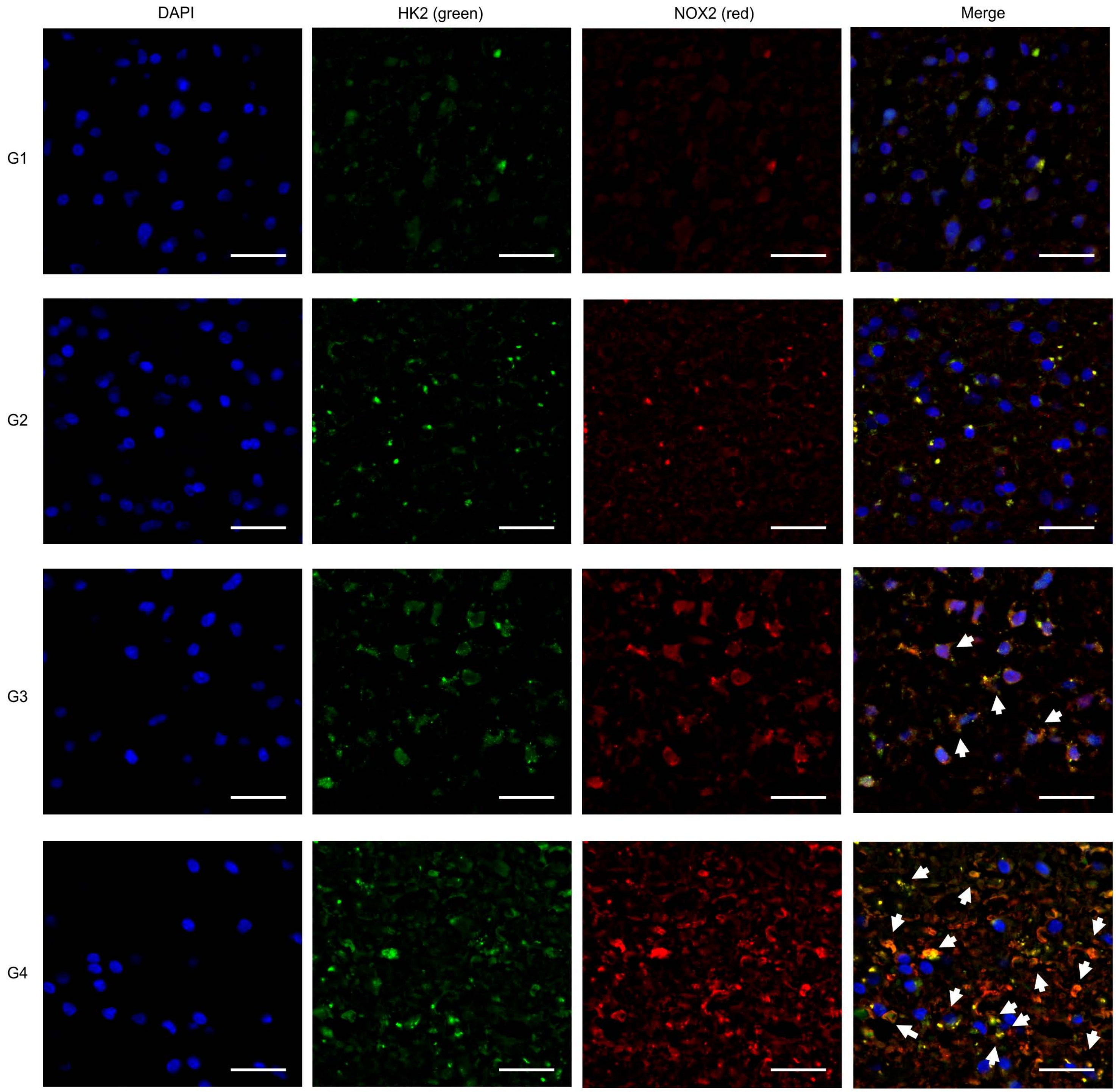


**Supplementary Figure S3.**

**The levels of NOX2 and HK2 were elevated in patients with GBM.** Representative immunofluorescence images of NOX2 and HK2 protein expression in tissues from patients with GBM (G4), G3 glioma (G3), G2 glioma (G2), and G1 glioma (G1) showing NOX2 (red) and HK2 (green) in glioma cells. DAPI-stained nuclei are shown in blue. Scale bars, 20 μM. White arrows indicate NOX2 and HK2 positive cells.

## Supplementary Figure S4

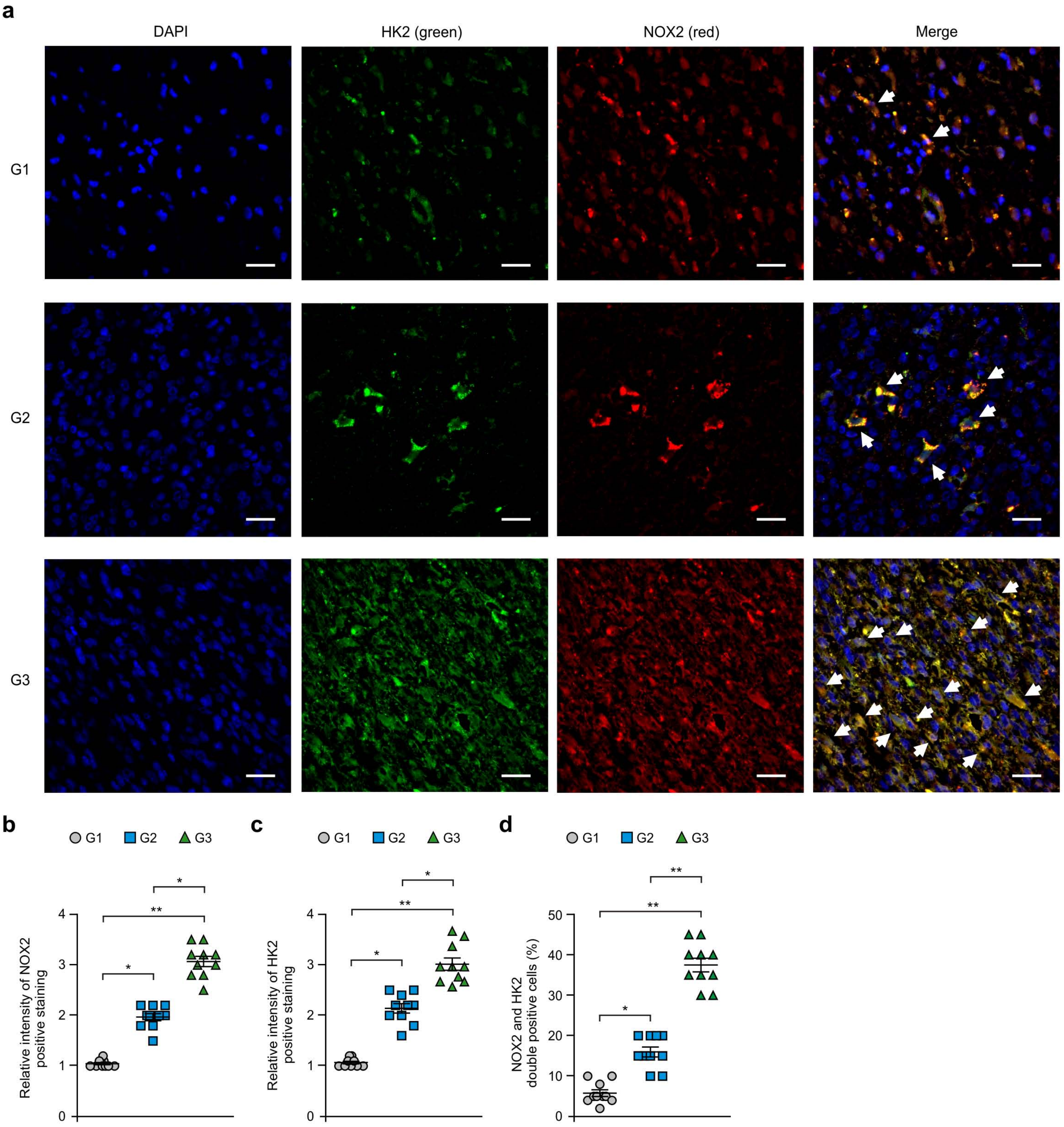


**Supplementary Figure S4.**

**The levels of NOX2 and HK2 were elevated in patients with high-grade glioma.** (a) Representative immunofluorescence images of NOX2 and HK2 protein expression in tissues from patients with G3 glioma (G3), G2 glioma (G2), and G1 glioma (G1) showing NOX2 (red) and HK2 (green) in glioma cells. DAPI-stained nuclei are shown in blue. Scale bars, 20 μM. White arrows indicate NOX2 and HK2 positive cells. (b) Relative intensity of NOX2 positive staining, (c, d) Relative intensity of HK2 positive staining (c) and quantification of NOX2 and HK2 positive glioma cells (d) from immunofluorescence images in tissues from patients with G3 glioma (G3), G2 glioma (G2), and G1 glioma (G1) (n = 10 per area of individual subject). Data are mean ± SD. **, $p < 0.01$; *, $p < 0.05$ by ANOVA or Student's two-tailed t-test.

## Supplementary Figure S5

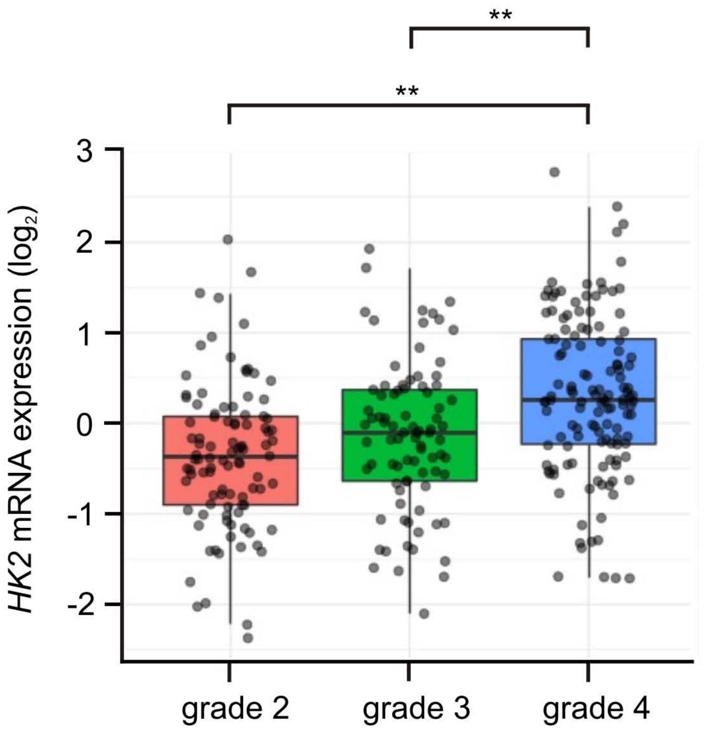


**Supplementary Figure S5.**

**Replication study for *HK2* gene expression with REMBRANDT glioma dataset to evaluate results from TCGA.**

The levels of *HK2* mRNA in patients with grade 2, grade 3 and grade 4 glioma. ** $p < 0.01$.

## Supplementary Figure S6

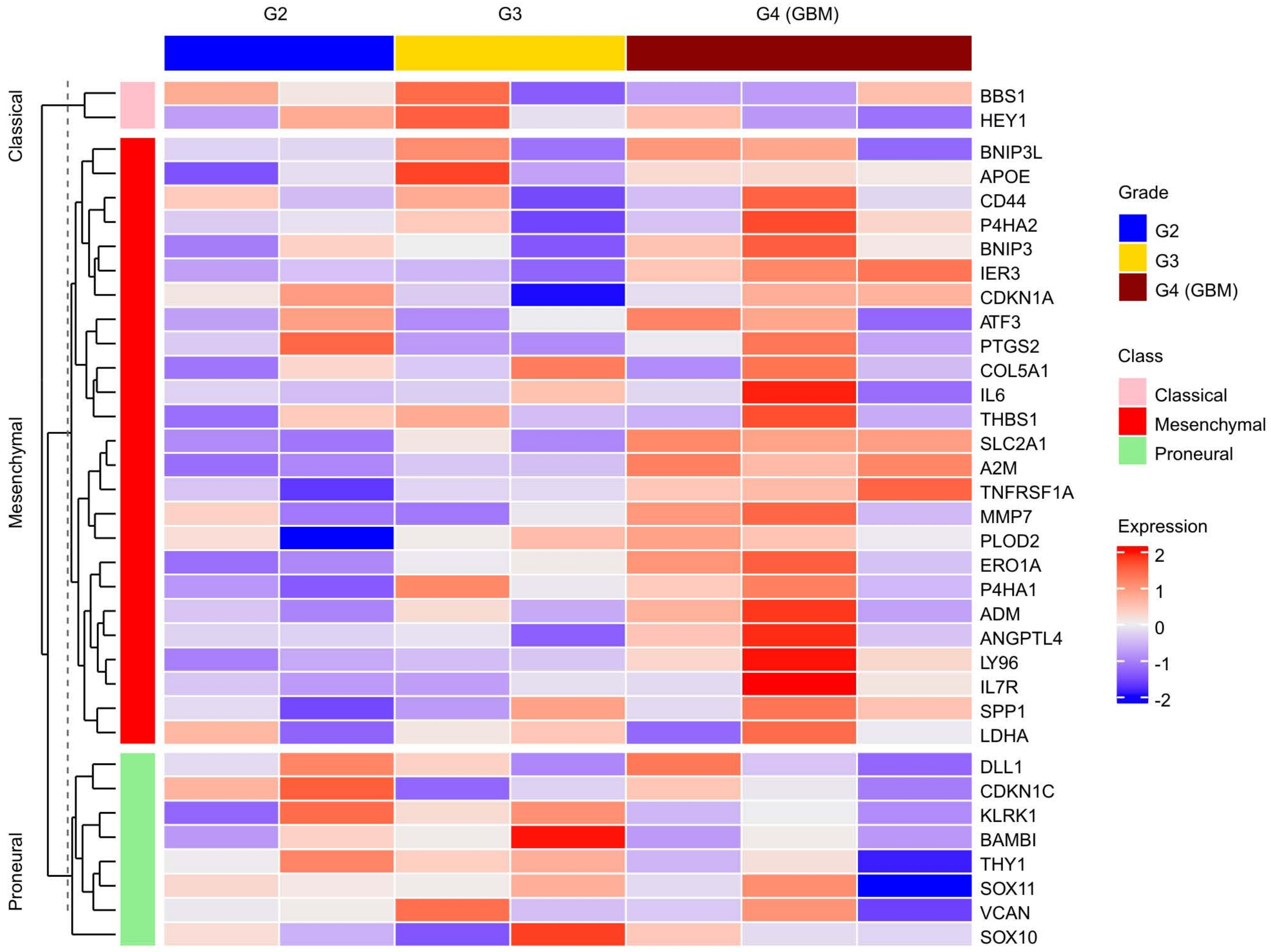


**Supplementary Figure S6.**

**The levels of mesenchymal subtype signature genes were elevated in glioma tissues of patients with GBM.**
Heatmaps illustrating for gene expression patterns of proneural stubtype genes, mesenchymal stubtype genes, and classical stubtype genes in glioma tissues of patients with GBM (G4), G3 glioma (G3), and G2 glioma (G2).

## Supplementary Figure S7

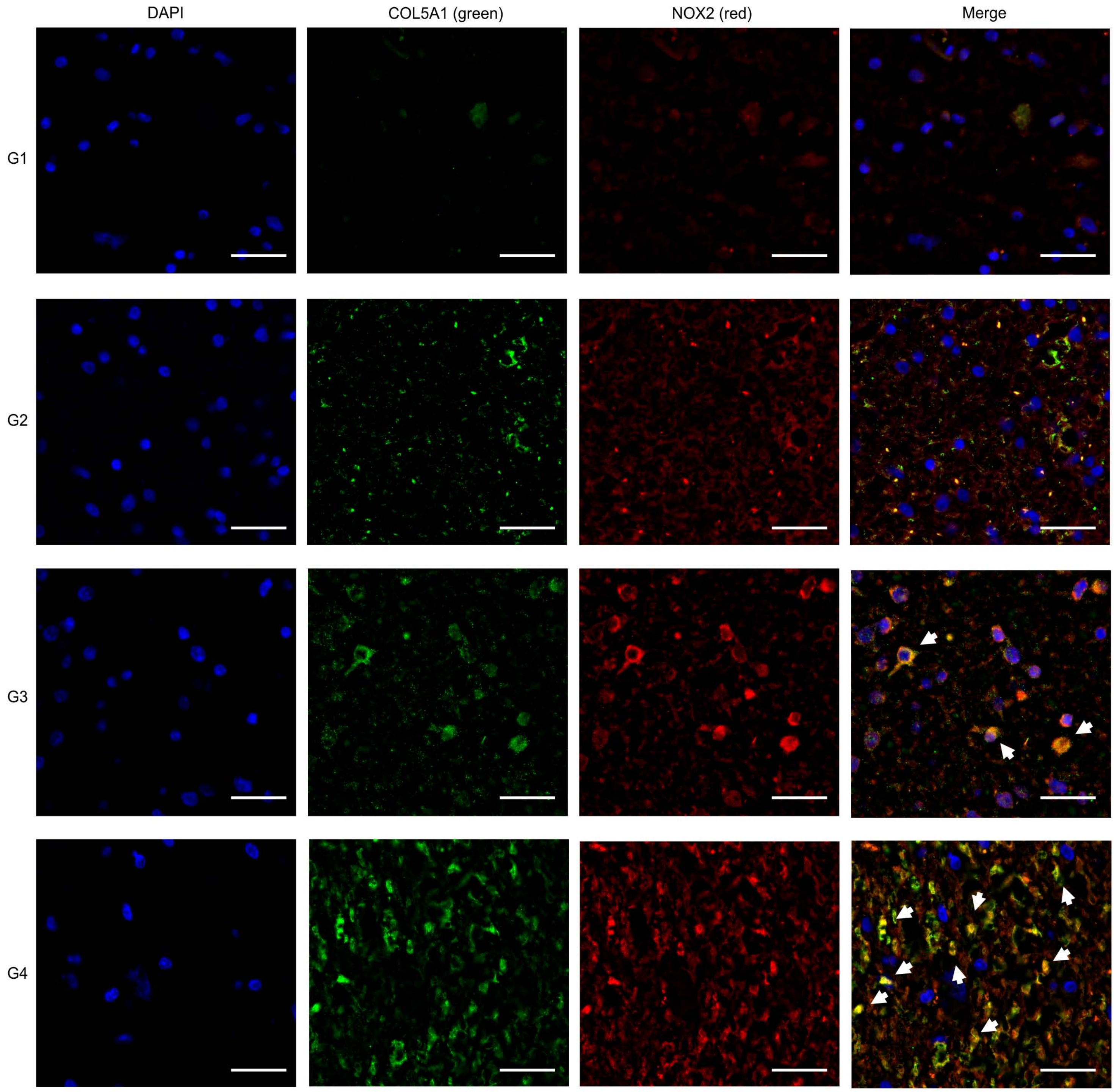


**Supplementary Figure S7.**

**The levels of NOX2 and COL5A1 were elevated in patients with GBM.** Representative immunofluorescence images of NOX2 and COL5A1 protein expression in tissues from patients with GBM (G4), G3 glioma (G3), G2 glioma (G2), and G1 glioma (G1) showing COL5A1 (green) and NOX2 (red) in glioma cells. DAPI-stained nuclei are shown in blue. Scale bars, 20 μM. White arrows indicate NOX2 and COL5A1 positive cells.

# Supplementary Figure S8

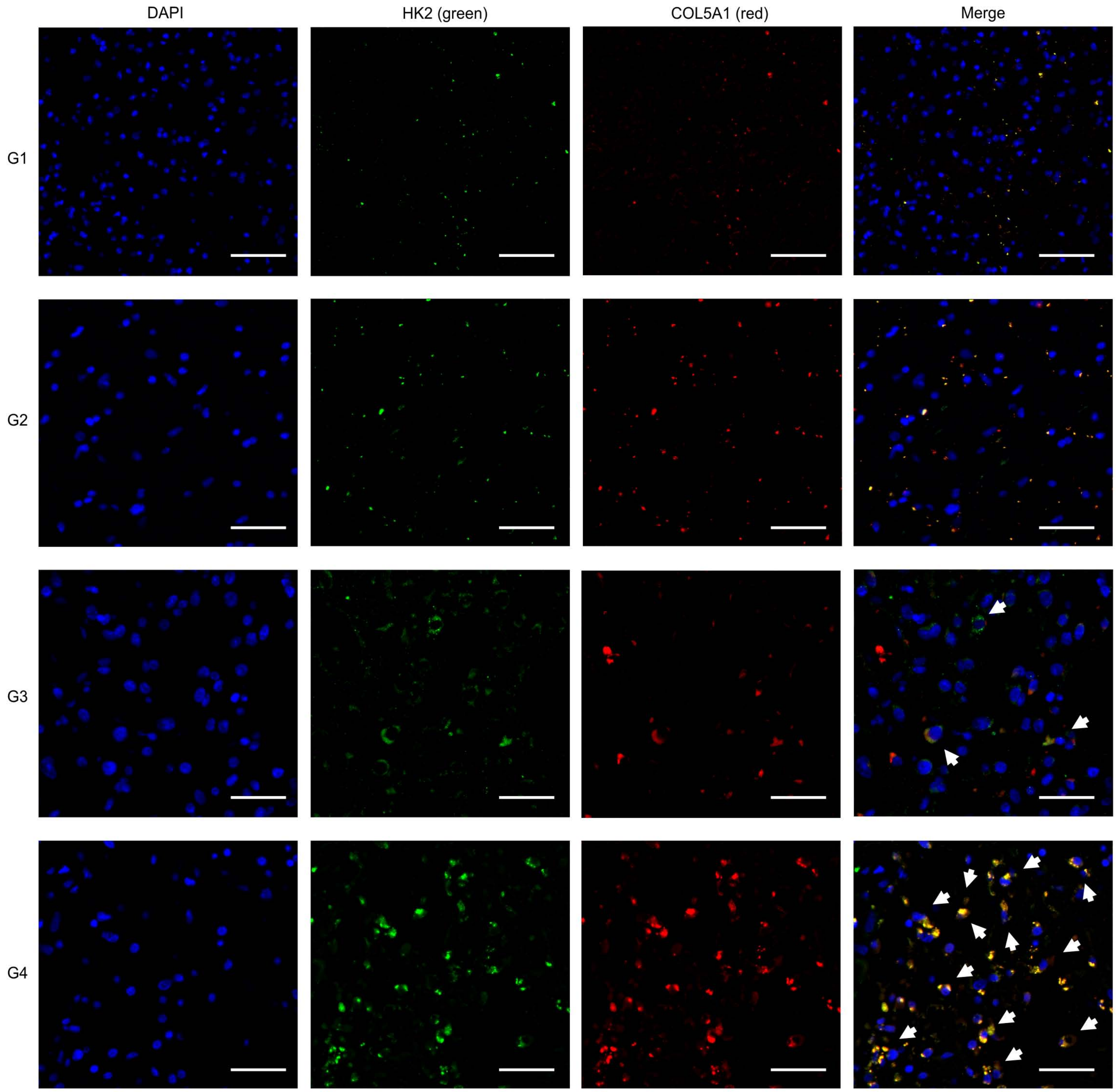


**Supplementary Figure S8.**

**The levels of HK2 and COL5A1 were elevated in patients with GBM.** Representative immunofluorescence images of HK2 and COL5A1 protein expression in tissues from patients with GBM (G4), G3 glioma (G3), G2 glioma (G2), and G1 glioma (G1) showing HK2 (green) and COL5A1 (red) in glioma cells. DAPI-stained nuclei are shown in blue. Scale bars, 20 μM. White arrows indicate NOX2 and COL5A1 positive cells.

## Supplementary Figure S9

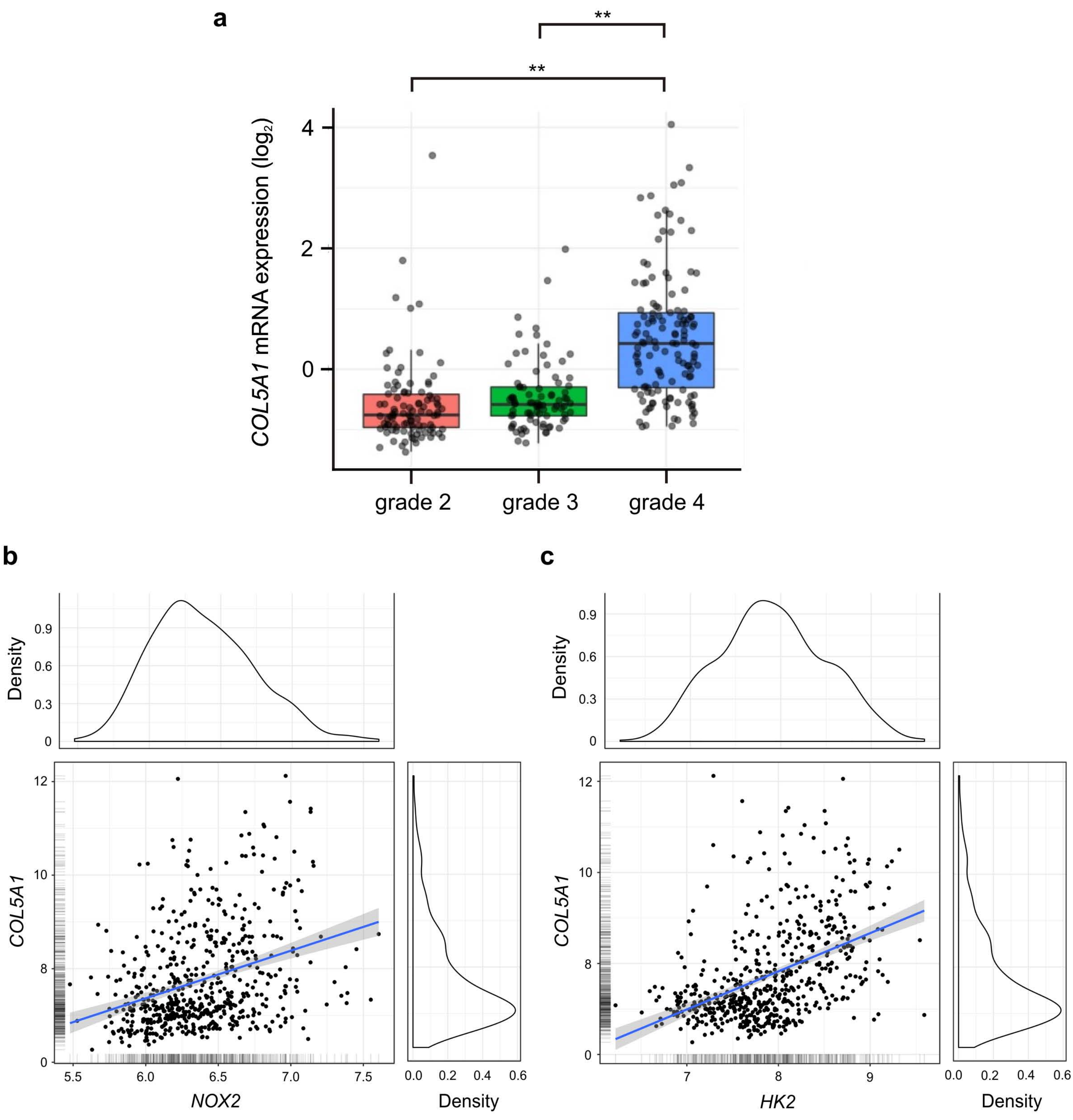


**Supplementary Figure S9.**

**Replication study for *COL5A1* gene expression with REMBRANDT glioma dataset to evaluate results from TCGA.** (a) The levels of *COL5A1* mRNA in patients with grade 2, grade 3 and grade 4 glioma. $^{**}p < 0.01$. (b) Correlation analysis between *NOX2* and *COL5A1* gene (Correlation coefficient : 0.34, $^{*}p < 0.05$) and (c) Correlation analysis between *HK2* and *COL5A1* gene (Correlation coefficient : 0.46, $^{*}p < 0.05$) from REMBRANT GBM and LGG dataset.
