# Supplementary figures and images for: NOX2-Induced High Glycolytic Activity Contributes to the Gain of COL5A1-Mediated Mesenchymal Phenotype in GBM
Source: Cancers (Basel). 2022 Jan 20;14(3):516. doi: 10.3390/cancers14030516 (PMC8833670; doi:10.3390/cancers14030516)

Figure 2

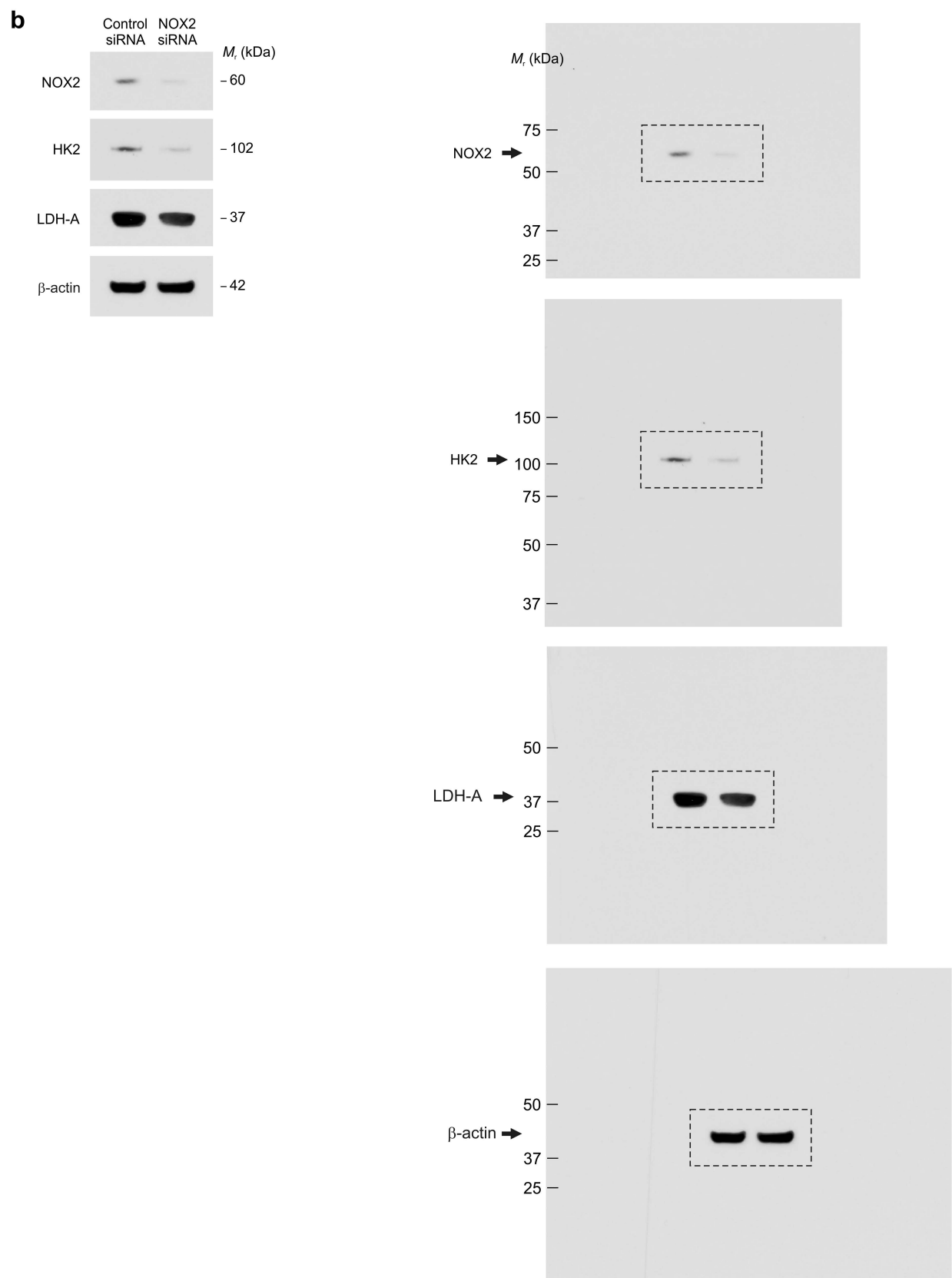

Figure 2

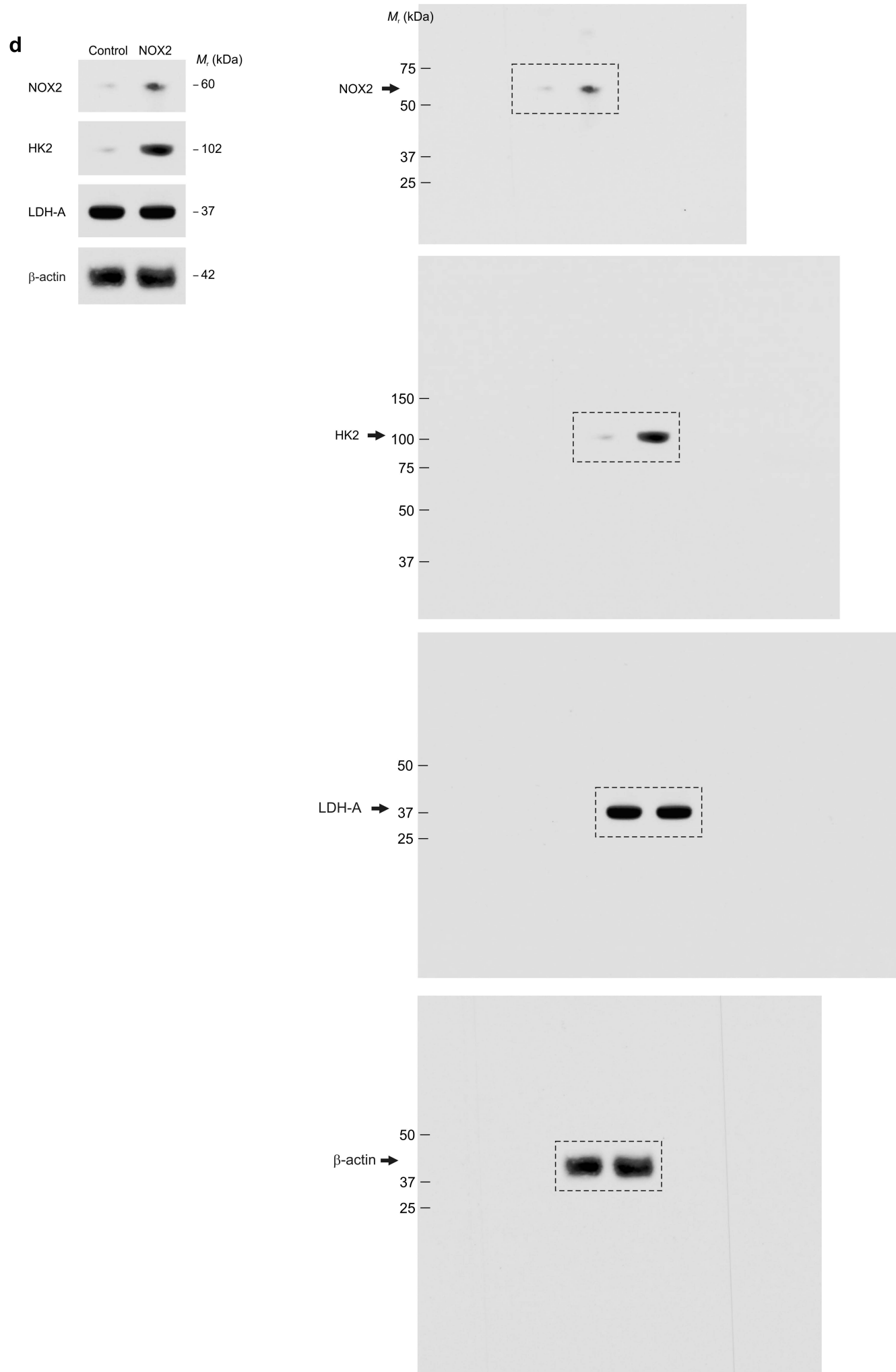

Figure 3

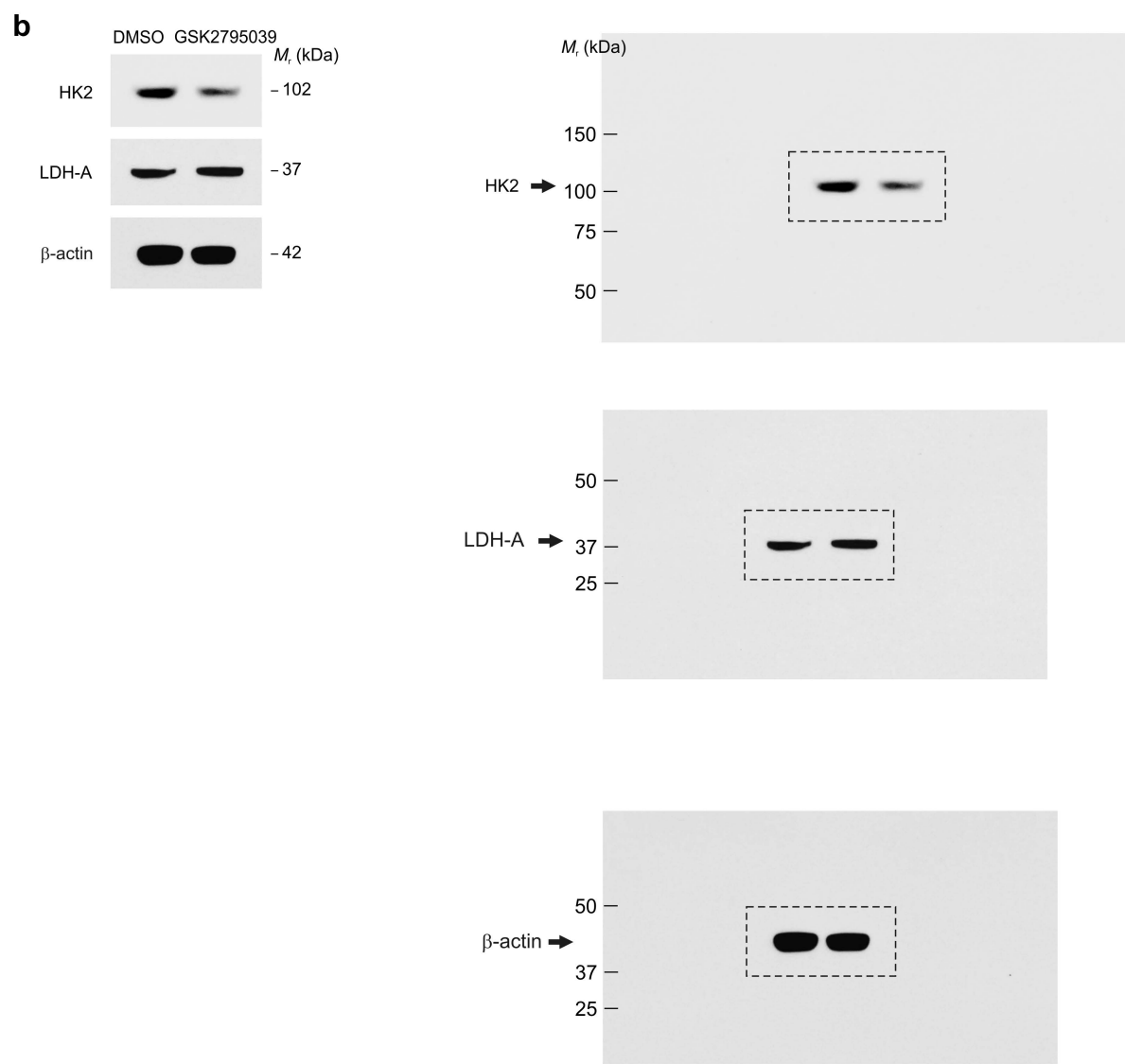

Figure 5

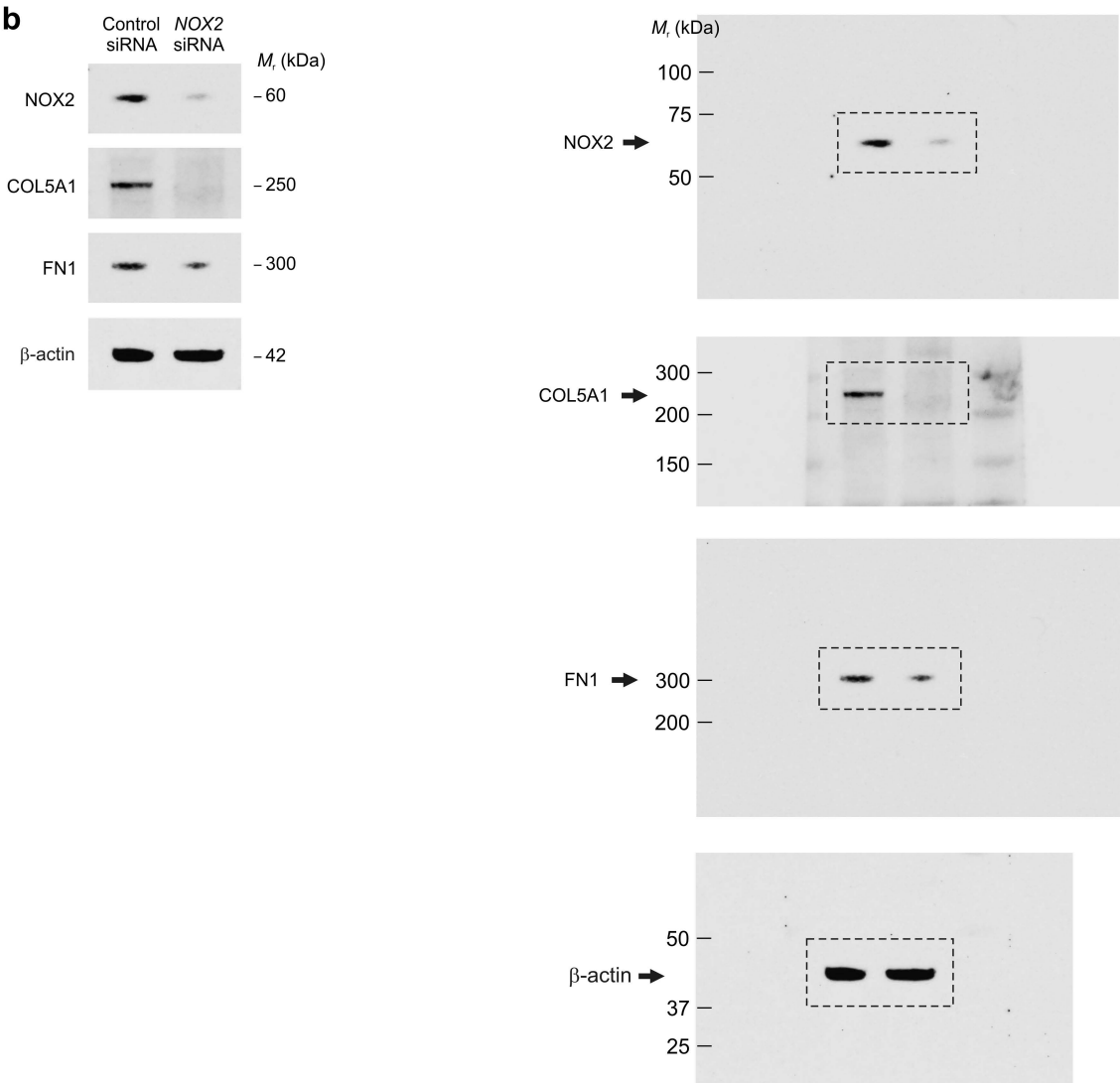

Figure 5

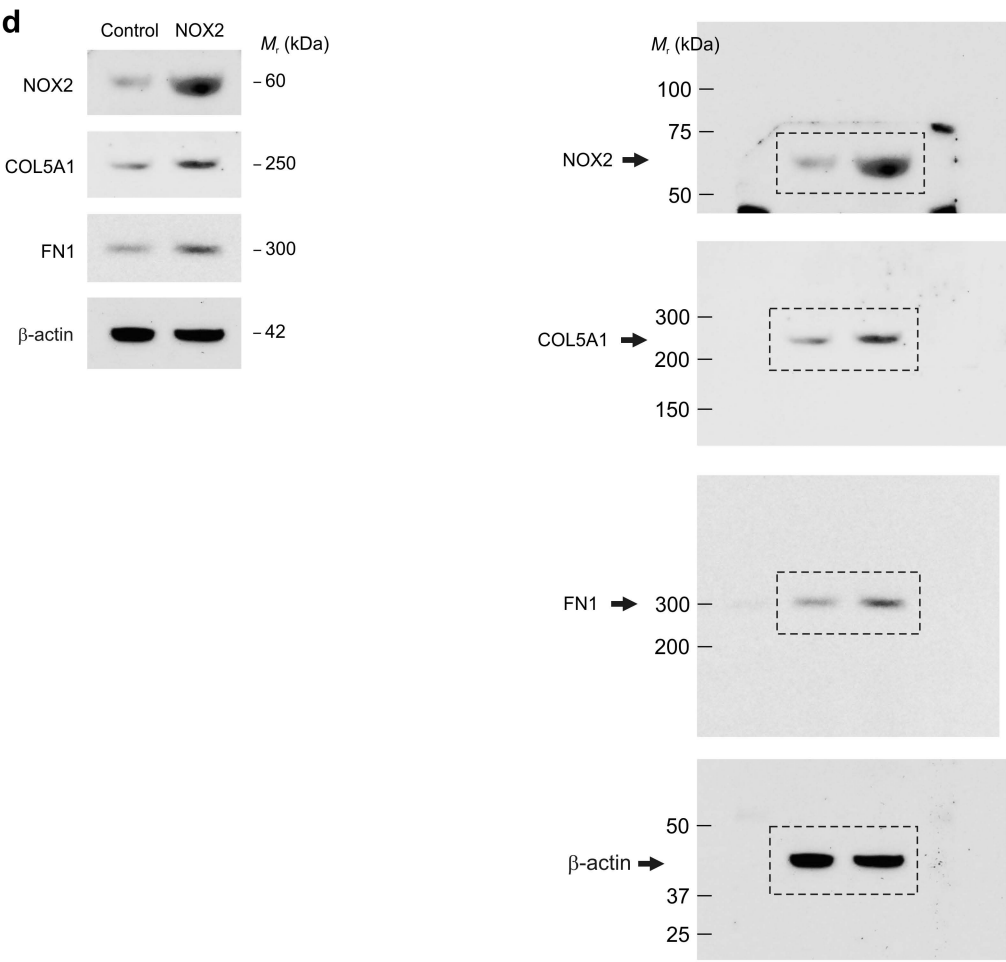

Figure 5

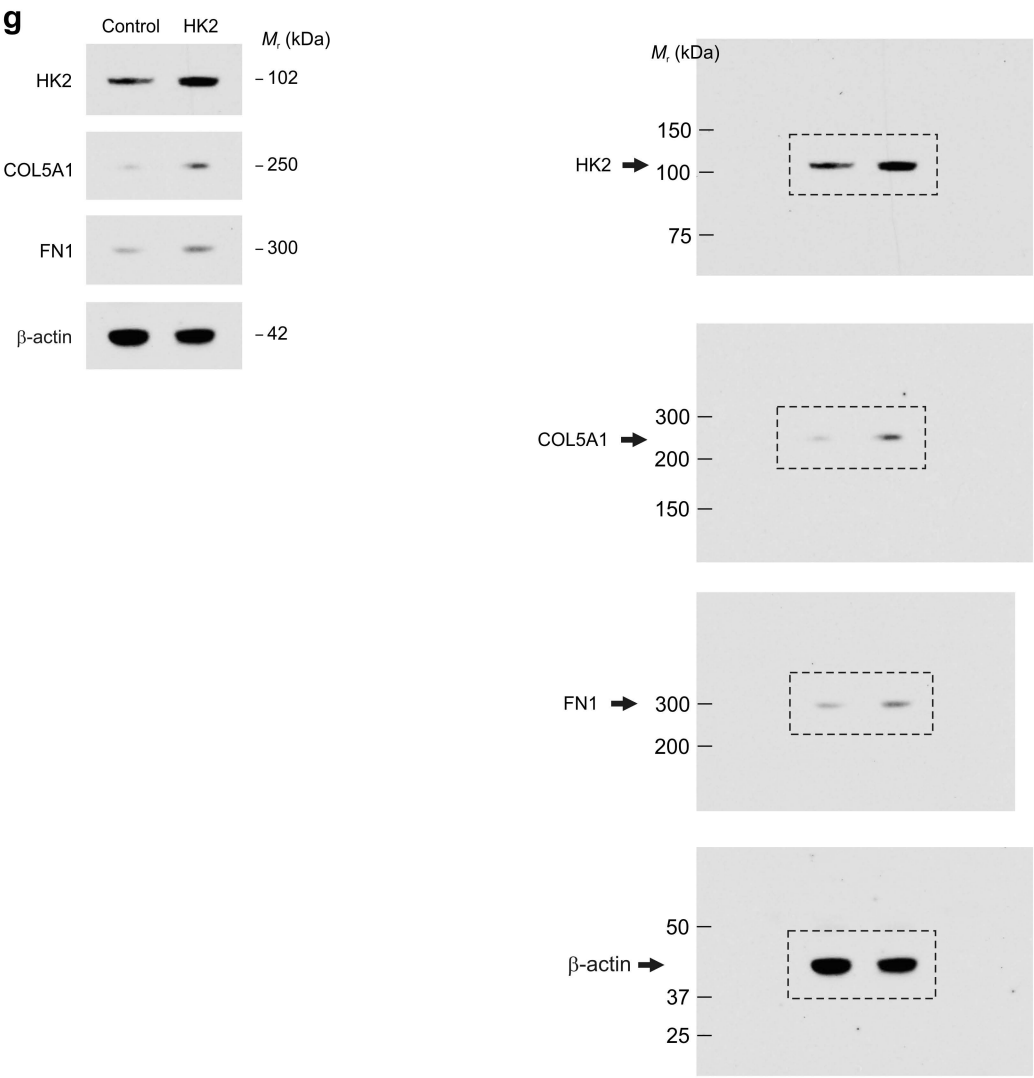

Supplement: Supplementary file 1 [file cancers-14-00516-s001.zip › cancers-1540096.pdf]
